# Expression patterns and immunological characterization of PANoptosis -related genes in gastric cancer

**DOI:** 10.3389/fendo.2023.1222072

**Published:** 2023-08-18

**Authors:** Xin Qing, Junyi Jiang, Chunlei Yuan, Kunke Xie, Ke Wang

**Affiliations:** ^1^ Clinical Laboratory, Boai Hospital of Zhongshan Affiliated to Southern Medical University, Zhongshan, China; ^2^ West China Hospital, Sichuan University, Chengdu, China

**Keywords:** PANoptosis, gastric cancer, molecular patterns, immune infiltration, machine learning

## Abstract

**Background:**

Accumulative studies have demonstrated the close relationship between tumor immunity and pyroptosis, apoptosis, and necroptosis. However, the role of PANoptosis in gastric cancer (GC) is yet to be fully understood.

**Methods:**

This research attempted to identify the expression patterns of PANoptosis regulators and the immune landscape in GC by integrating the GSE54129 and GSE65801 datasets. We analyzed GC specimens and established molecular clusters associated with PANoptosis-related genes (PRGs) and corresponding immune characteristics. The differentially expressed genes were determined with the WGCNA method. Afterward, we employed four machine learning algorithms (Random Forest, Support Vector Machine, Generalized linear Model, and eXtreme Gradient Boosting) to select the optimal model, which was validated using nomogram, calibration curve, decision curve analysis (DCA), and two validation cohorts. Additionally, this study discussed the relationship between infiltrating immune cells and variables in the selected model.

**Results:**

This study identified dysregulated PRGs and differential immune activities between GC and normal samples, and further identified two PANoptosis-related molecular clusters in GC. These clusters demonstrated remarkable immunological heterogeneity, with Cluster1 exhibiting abundant immune infiltration. The Support Vector Machine signature was found to have the best discriminative ability, and a 5-gene-based SVM signature was established. This model showed excellent performance in the external validation cohorts, and the nomogram, calibration curve, and DCA indicated its reliability in predicting GC patterns. Further analysis confirmed that the 5 selected variables were remarkably related to infiltrating immune cells and immune-related pathways.

**Conclusion:**

Taken together, this work demonstrates that the PANoptosis pattern has the potential as a stratification tool for patient risk assessment and a reflection of the immune microenvironment in GC.

## Introduction

Regulated cell death (RCD) is mediated by multiple signal transduction pathways and presented a clear action mechanism in the pathophysiologic process (1, 2). As non-apoptosis RCD forms, autophagy, ferroptosis, pyroptosis, and necroptosis have been observed to serve a crucial role in the maintenance of homeostasis and disease progression (3–5). Meanwhile, A growing insight into the interaction between pyroptosis, apoptosis, and necroptosis has contributed to the aggregation of these three RCD modalities into one concept: PANoptosis (6, 7). Once an RCD pathway is terminated in the tumor, PANoptosis can immediately initiate an alternative mechanism to act as a tumor suppressor (8). Several reports have confirmed that PANoptosis drives the development of a variety of diseases such as colorectal cancer, ARDS, and ischemia injury (9–11). Although numerous reports have revealed the importance of pyroptosis, apoptosis, and necroptosis respectively in tumors, the relationship between PANoptosis and antitumor immunity remains unknown. Identifying the PANoptosis-related molecular mechanisms can provide potential opportunities to generate promising insights for tumor immunotherapy.

Gastric cancer (GC) is one of the most prevalent magnificence in the digestive system (12). GC at an early stage has an excellent prognosis with a 5-year survival probability of over 90%, while the 5-year survival probability of progressive GC is only about 30% (13, 14). Early management of GC is crucial to improve the survival status and reduce the mortality probability of patients, and it is essential to precisely identify GC with specific molecular patterns and establish a multivariate prediction signature (15, 16). Meanwhile, growing evidence from the multi-omics advancement enables us to access a landscape insight into RCDs in GC (17–19). Therefore, additional studies on the molecular levels of PANoptosis-related genes (PRGs) may offer a novel perspective into GC heterogeneity.

In this study, we investigated gene expression and immune landscape differences between control and GC specimens. GC patients were categorized into two PANoptosis-related clusters, and cluster-specific DEGs were determined with the WGCNA algorithm. Subsequently, a predictive model for classifying patients with distinct molecular patterns and evaluating its reliability with various methods and external validation cohorts, thereby offering promising perspectives into the prediction of GC patterns and risk.

## Materials and methods

### Data collection and preprocessing

The available data were obtained from the Gene Expression Omnibus (GEO) public database (20), including GSE54129, GSE65801, GSE66229, and GSE13911. These datasets focused on the gene sequencing results of GC patients, and each dataset contains more than 60 samples (**Table 1**). GSE54129 and GSE65801 were further integrated, and the batch effects between different reports and platforms were eliminated with the combat algorithm in the sva package (21). The integrated dataset was applied for further analysis, and GSE66229 and GSE13911 were regarded as external validation cohorts.

**Table 1:** Information on microarray datasets obtained from GEO database.

Dataset	Platform	GC	Control
GSE54129	GPL570	111	21
GSE65801	GPL14550	32	32
GSE66229	GPL570	300	100
GSE13911	GPL570	31	38

### Correlation analysis between PRGs and immune infiltration analysis

14 PRGs were acquired from the prior report (22), and 10 genes were identified as differentially expressed genes. The CIBERSORT algorithm was further utilized to evaluate the abundant levels of 22 kinds of immune cells in individual samples (23). To reveal the relationship between PRGs and GC-related immunological features, we explored the correlation between the PRGs and the abundance of immune cells. p < 0.05 indicated a statistical significance.

### Unsupervised clustering of GC patients

Based on these PRGs’ expression data, the unsupervised clustering analysis (“ConsensusClusterPlus” package) categorized the 143 GC samples into distinct clusters with the k-means algorithm for 1,000 iterations (24). The optimal cluster variable was systematically assessed based on the cumulative distribution function (CDF) curve, consensus matrix, and consistent cluster score (>0.9).

### Weighted gene co-expression network analysis (WGCNA)

WGCNA is a bioinformatics approach for introducing patterns of gene correlations between different specimens and revealing gene module information with biological significance (25, 26). First, the correlation parameter between paired genes was computed to establish the correlation matrix. Next, this matrix was transferred into a weighted neighborhood matrix based on the soft threshold feature. Afterward, the neighborhood matrix was further converted into a topological overlap matrix (TOM) revealing the correlative levels between genes. 1-TOM was regarded as the distance for clustering the genes, and the dynamic tree chopping was constructed to determine the module. The least gene number in the modules was set to 100. After the individual module was identified according to the key gene expression data and the sample classification, the association of the module key genes with sample classifications was also identified.

### Establishment of the prediction model based on machine learning algorithms

Based on two distinct PRGs clusters, the “caret” package was utilized to propose machine learning models including random forest model (RF), support vector machine model (SVM), generalized linear model (GLM), and eXtreme Gradient Boosting (XGB) (27). According to various dependent decision trees from a training pool, the RF algorithm promotes the precision of the model by randomly limiting the overfitting of individual decision trees (28). SVM can identify optimal parameters by removing the SVM-derived eigenvectors (29). An SVM module based on the “e1071” package was created to further evaluate the diagnostic value of the selected biomarker in GC (30). GLM could dynamically assess the association between normally distributed dependent traits and categorical or continuous independent traits (31). XGB is a collection of boosted trees based on gradient boosting, thus making detailed comparability between classification error and model sophistication (32). The different clusters were regarded as the response parameter and the cluster-specific DEGs were identified as interpretative parameters. The caret package dynamically adjusted the variables in these models by grid identification, and these machine learning models were executed with default factors and evaluated with 5-fold cross-validation. The “DALEX” package was performed to illustrate the abovementioned four machine learning models and visualize the residual distribution and feature importance among these machine learning models (33). The “pROC” package was executed to quantify the area under ROC curves (34). Finally, the appropriate machine learning model was identified and the top 5 parameters were regarded as the crucial predictive indicators correlated with GC. The predictive model was confirmed for its diagnostic value using ROC curves analysis in the validation datasets.

### Diagnostic nomogram construction and validation

A diagnostic nomogram was obtained for the risk evaluation of GC with the “rms” package (35). Nomogram presented individual risk values for individual variables; a final value was acquired by merging the value of 5 selected variables. Afterward, the risk of GC could be obtained based on the final value. The diagnostic reliability of the nomogram was evaluated with the calibration curve, DCA, and external cohorts.

### Correlation between selected variables and infiltrating immune cells

Based on the CIBERSORT algorithm, correlation evaluation was executed to investigate the association between infiltrating immune cells and selected variables. These results were visualized with the “ggplot2” package (36). P-values < 0.05 demonstrated statistical significance. Meanwhile, immune-check points and HLA molecules were also discussed in immunological analysis.

## Results

### Dysregulation of PANoptosis regulators and immunological status in GC

**Figure 1:**
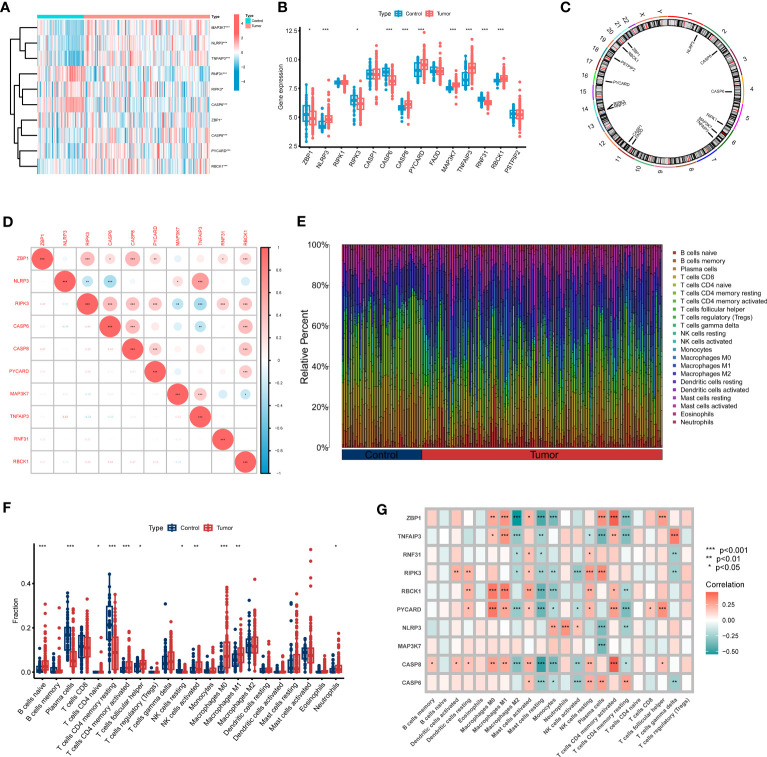
Identification of dysregulated PRGs in GC. **(A)** The expression landscape of 10 DEGs was presented in the heatmap. **(B)** Boxplots illustrated the expression of 14 PRGs between GC and control samples. **(C)** The location of 14 PRGs on chromosomes. **(D)** Correlation analysis of 10 differentially expressed PRGs. **(E)** The relative abundances of 22 infiltrating immune cells between GC and control samples. **(F)** Boxplots showed the differences in immune infiltration between GC and control samples. **(G)** Correlation analysis between 10 differentially expressed PRGs and infiltrating immune cells. *p < 0.05, **p < 0.01, ***p < 0.001.

To explore the molecular activity of PANoptosis modulators in the development of GC, we comprehensively assessed the abundant levels of 14 PRGs between GC and control samples using the merged dataset. 10 PRGs were identified as the differentially expressed PANoptosis genes. The relative levels of ZBP1, RIPK3, CASP6, and RNF31 were lower, whereas NLRP3, CASP8, PYCARD, MAP3K7, TNFAIP3, and RBCK1 were significantly higher in GC than that in control samples (**Figures 1A, B**). **Figure 1C** presents the site of CNV alterations of these regulators on chromosomes. Afterward, we conducted a correlation examination between these differentially expressed PRGs to investigate whether PANoptosis modulators serve a vital function in the evolution of GC. Interestingly, a significant synergistic impact was observed in most of the regulators, such as NLRP3 and TNFAIP3 (coefficient = 0.63) (**Figure 1D**).

To clarify whether there are immunological differences between the GC and control samples, the CIBERSORT method was executed to identify the enrichment difference of immune infiltration cells between GC and control samples (**Figure 1E**). The findings revealed that GC patients demonstrated greater enrichment levels of naïve B cells, naïve CD4 T cells, activated memory CD4 T cells, follicular helper T cells, activated NK cells, M0 Macrophages, M1 Macrophages, and Neutrophils (**Figure 1F**), indicating that variations in the immunological status could serve a critical role in the progression of GC. Additionally, we also explored the association between differentially expressed PANoptosis regulators and infiltrating immune cells. The majority of immune cells were markedly associated with these regulators (**Figure 1G**). These findings demonstrated that PRGs may be the vital variables in modulating the molecular and immunological landscape of GC patients.

### Immunological features of PANoptosis clusters

**Figure 2:**
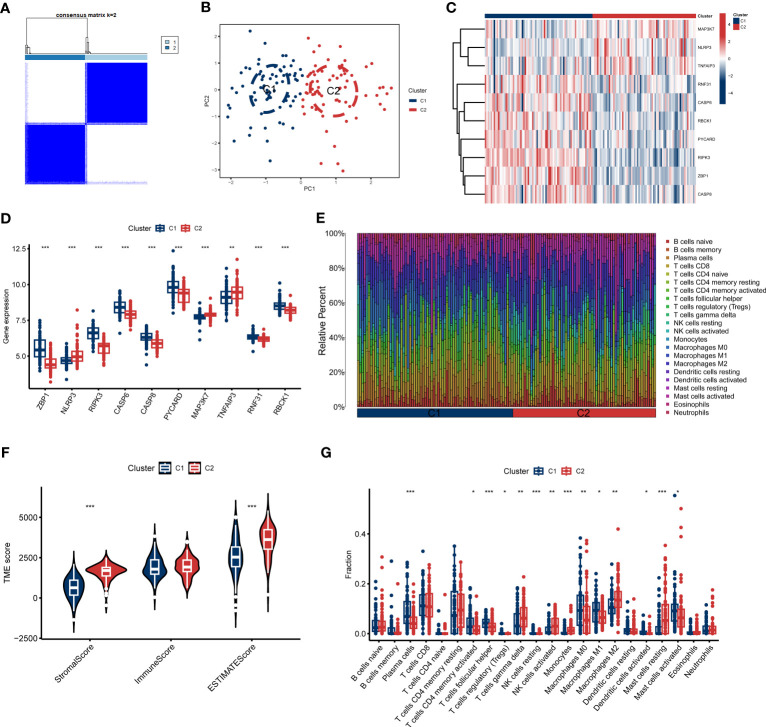
Identification of molecular and immune characteristics between the two PANoptosis clusters. **(A)** Consensus clustering matrix when k = 2. **(B)** PCA visualizes the distribution of two clusters. **(C, D)** Expression landscape of 10 DEGs between two PANoptosis clusters. **(E)** The relative levels of 22 infiltrating immune cells between two clusters. **(F)** Estimated immune microenvironment scores between the two clusters. **(G)** The differences in immune infiltration between two clusters. *p < 0.05, **p < 0.01, ***p < 0.001.

To clarify the PANoptosis-related patterns in GC, we categorized the GC samples from the expression status of 10 differentially expressed PRGs. The k-value was adjusted to 2 (**Figure 2A**), and GC patients were finally classified into two clusters, including Cluster1 (n = 73) and Cluster2 (n = 70). The findings of the principal component analysis (PCA) analysis confirmed that there was a remarkable discrepancy between these clusters (**Figure 2B**).

To further investigate the molecular properties of clusters, we fully discussed the enrichment differences of 10 PRGs between clusters. Different PRGs expression patterns were clarified between the two PANoptosis clusters (**Figure 2C**). PANoptosis Cluster1 was typified by elevated expressions of ZBP1, RIPK3, CASP, CASP8, PYCARD, TNFAIP3, RNF31, and RBCK1, while PANoptosis Cluster2 presented high expression levels of NLRP3 and MAP3K7 (**Figure 2D**). Similarly, the immune infiltration analyses indicated a distinct immune microenvironment between the two clusters (**Figure 2E**). Cluster1 demonstrated decreased Stromal score and ESTIMATE score, consistent with the prior results (**Figure 2F**). Specifically, Cluster1 presented a greater ratio of Plasma cells, activated CD4 memory T cells, follicular helper T cells, Tregs, resting NK cells, M0 Macrophages, M1 Macrophages, activated Dendritic cells, and activated Mast cells, whereas the infiltration of γδ T cells, activated NK cells, Monocytes, M2 Macrophages, and resting Mast cells were relatively higher in Cluster2 (**Figure 2G**). Furthermore, we also discussed additional immunogenic signatures to identify the immunological characteristics of these clusters. As demonstrated in **Figure S2**, diverse immune-checkpoint molecules and HLA molecules were differently expressed in these clusters. These abovementioned findings suggested that PANoptosis Cluster1 may exhibit a more predominant degree of immune abundance.

### Construction of gene modules and co-expression network

**Figure 3:**
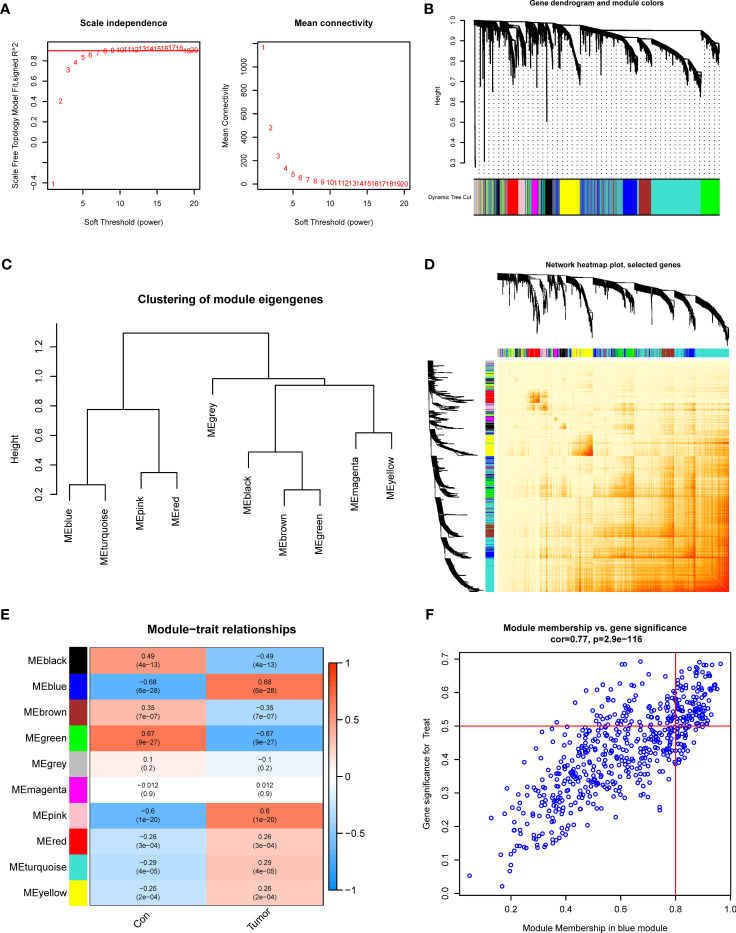
Co-expression network of DEGs in GC. **(A)** The identification of soft threshold power. **(B)** Cluster tree dendrogram of co-expression modules. **(C)** Representative of clustering of module eigengenes. **(D)** Representative heatmap of the correlations among 10 modules. **(E)** Correlation analysis between module eigengenes and clinical status. **(F)** Scatter plot between module membership in blue module and the gene significance for GC.

To determine the critical gene modules linked to GC, the WGCNA algorithm was utilized to construct a co-expression network and modules for the control and GC samples. We obtained the variance of gene expression in the merged dataset and identified the top 25% of genes with the greatest variance for additional analysis. Co-expressed gene modules were established when the value of soft power was 6 and the scale-free R2 was set to 0.9 (**Figure 3A**). A total of 10 specific

Co-expression modules with distinct colors were identified with the dynamic cutting approach and the heatmap of the topological overlap matrix (TOM) was also displayed (**Figures 3B–D**). Afterward, all genes in the 10 modules were further utilized for determining the similarity and adjacency of module-clinical characteristics (Control and GC) co-expression. Finally, the blue module presented the greatest connection with GC, which included 280 genes (**Figure 3E**). Meanwhile, we found a close relationship between the blue module and module-related genes (**Figure 3F**).

**Figure 4:**
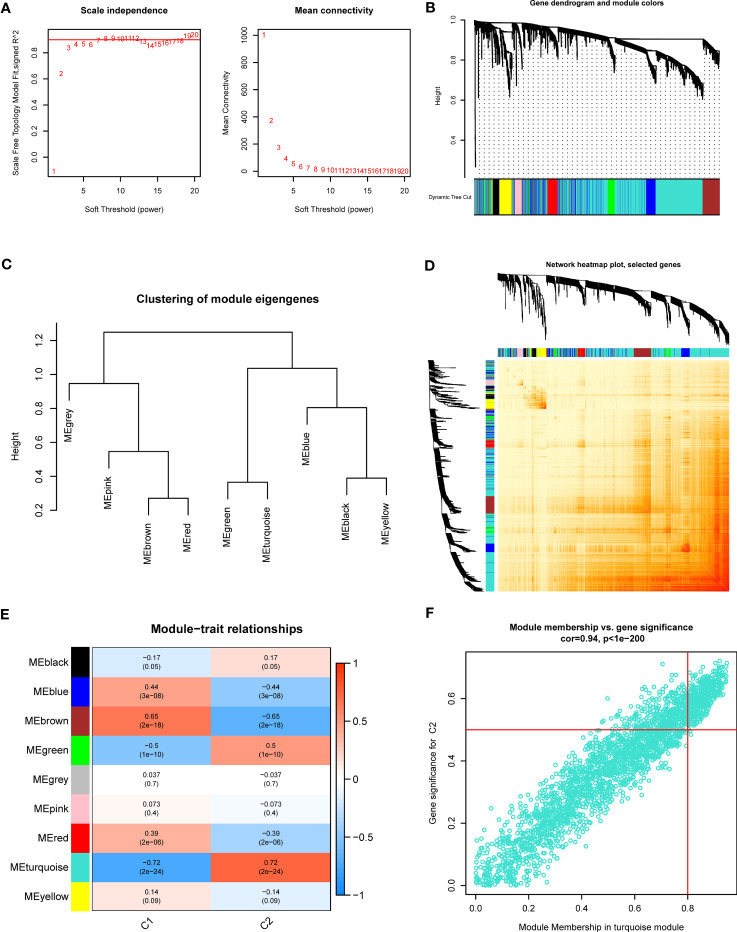
Co-expression network of DEGs between the two PANoptosis clusters. **(A)** The identification of soft threshold power. **(B)** Cluster tree dendrogram of co-expression modules. **(C)** Representative of clustering of module eigengenes. **(D)** Representative heatmap of the correlations among 9 modules. **(E)** Correlation analysis between module eigengenes and clinical status. **(F)** Scatter plot between module membership in turquoise module and the gene significance for Cluster2.

Additionally, we also investigated the vital gene modules associated with PANoptosis clusters with the WGCNA approach. We selected β = 4 and R2 = 0.9 as the optimal soft threshold variables to establish a scale-free network (**Figure 4A**). More critically, 9 modules were identified as valuable modules and the heatmap portrayed the TOM of each module-related gene (**Figures 4B–D**). Module-clinical characteristics (Cluster1 and Cluster2) relationship indicated the significant association between the turquoise module (750 genes) and GC clusters (**Figures 4E, F**). The findings demonstrated that turquoise module genes had a tight correlation with the screened module.

### Selection of cluster-specific DEGs and functional enrichment

**Figure 5:**
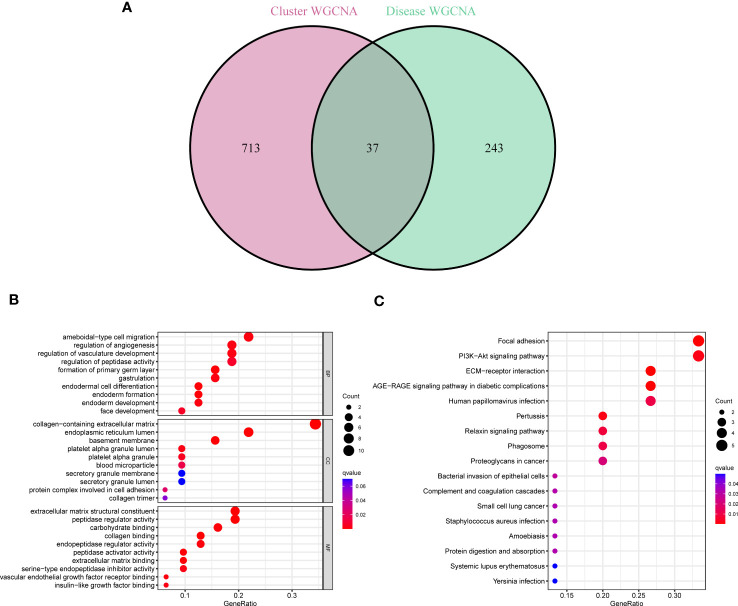
Identification of cluster-specific DEGs and their biological functions. **(A)** The intersections between module-related genes of PANoptosis clusters and module-related genes. **(B, C)** Functional enrichment analysis of overlapping genes based on GO and KEGG pathways.

37 cluster-specific DEGs were found by exploring the overlapping genes between module-related genes of PANoptosis clusters and module-related genes of GC and control samples (**Figure 5A**). The functional enrichment analysis was applied to investigate the functional annotations correlated with cluster-specific DEGs. The findings revealed that angiogenetic status and endoderm cell activity were closely associated with these DEGs based on GO pathways (**Figure 5B**). Meanwhile, we found these DEGs participated in diverse signaling processes, such as focal adhesion, PI3K-Akt signaling pathway, and ECM-receptor interaction (**Figure 5C**). Meanwhile, The GSVA analysis was applied to further investigate the functional differences related to cluster-specific DEGs between the two clusters (**Figure S1**).

### Establishment and evaluation of machine learning models

**Figure 6:**
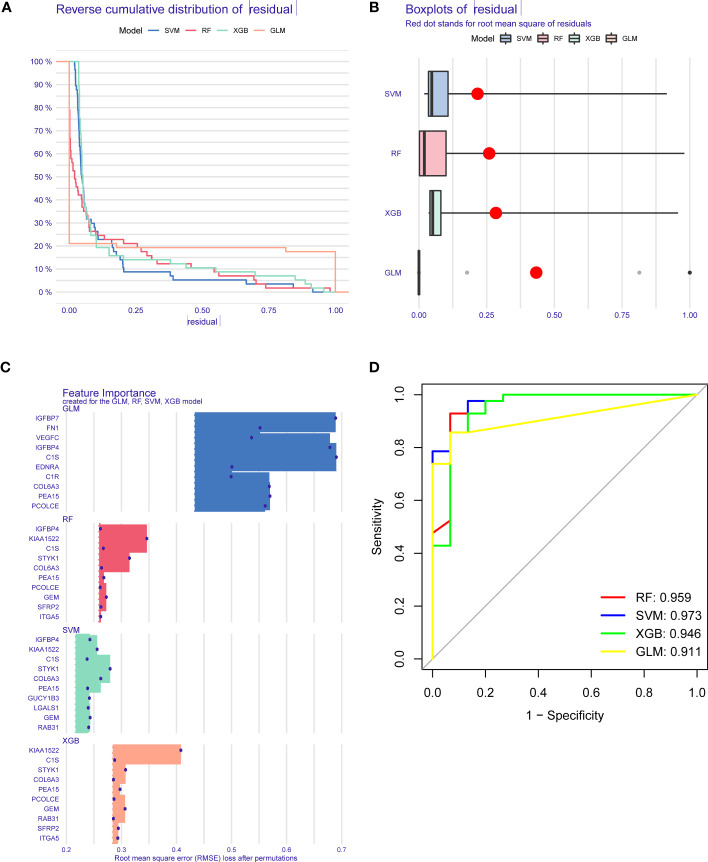
Establishment and evaluation of RF, SVM, GLM, and XGB machine models. **(A)** Cumulative residual distribution of each machine learning model. **(B)** Boxplots showed the residuals of each machine learning model. Red dot represented the root mean square of residuals (RMSE). **(C)** The important traits in RF, SVM, GLM, and XGB machine models. **(D)** ROC analysis of four machine learning models based on 5-fold cross-validation in the testing cohort.

To further determine pattern-specific genes with excellent diagnostic significance, we performed four machine learning algorithms (RF, SVM, GLM, and XGB) based on the expression data of 37 cluster-specific DEGs in the GC training cohort. The “DALEX” package was utilized to present the four algorithms and show the residual distribution of all models in the validation cohort. SVM and RF machine learning characteristics demonstrated a relatively smaller residual (**Figures 6A, B**). Afterward, the top 10 characteristic genes of the individual model were ranked based on the root mean square error (RMSE) (**Figure 6C**). Meanwhile, the receiver operating characteristic (ROC) curves were plotted to reveal the reliable status of these models, and we observed that SVM had the greatest performance (**Figure 6D**). Finally, the SVM model was presented to best distinguish patients with distinct patterns, and the top five critical genes (IGFBP4, GEM, KIAA1522, COL6A3, and STYK1) were identified as predictor variables for additional analysis.

**Figure 7:**
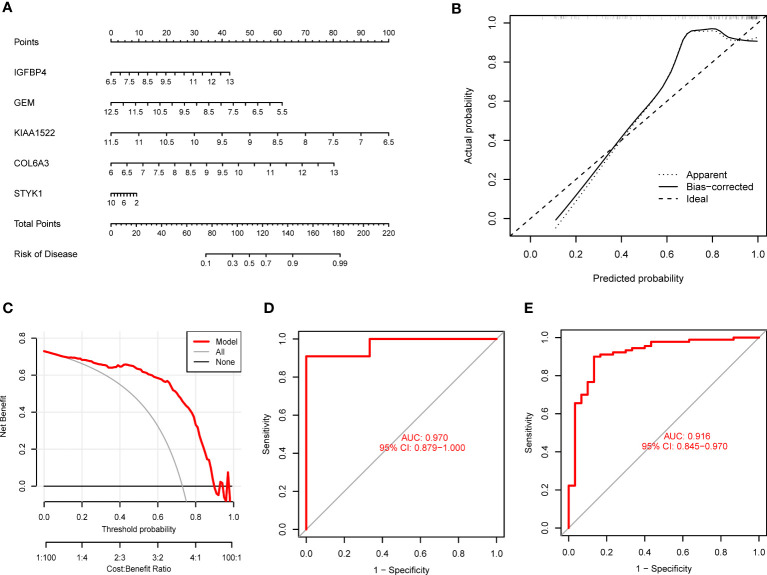
Validation of the 5-gene-based SVM model. **(A)** Establishment of a nomogram for predicting the risk of GC clusters based on the SVM model. **(B, C)** Construction of calibration curve **(B)** and DCA **(C)** for assessing the predictive efficiency of the nomogram model. **(D, E)** ROC analysis of the model based on 5-fold cross-validation in GSE13911 **(D)** and GSE66229 **(E)** datasets.

To validate the predictive reliability of the SVM model, we established a nomogram to assess the risk of PANoptosis patterns in GC patients (**Figure 7A**). The calibration curve further presented the predicted and actual pattern risks of GC (**Figure 7B**), and the DCA demonstrates that this nomogram

has excellent accuracy (**Figure 7C**), which may guide clinical decision-making. Subsequently, we confirmed the 5-gene predictive model on the external datasets (GSE13911 and GSE66229) including control samples and GC patients. ROC curves presented excellent performance of the 5-gene predictive model with an AUC value of 0.970 and 0.916, respectively (**Figures 7D, E**), suggesting this diagnostic model is equally valuable in distinguishing GC from normal samples.

### The biological activity and immune landscape of biomarkers

**Figure 8:**
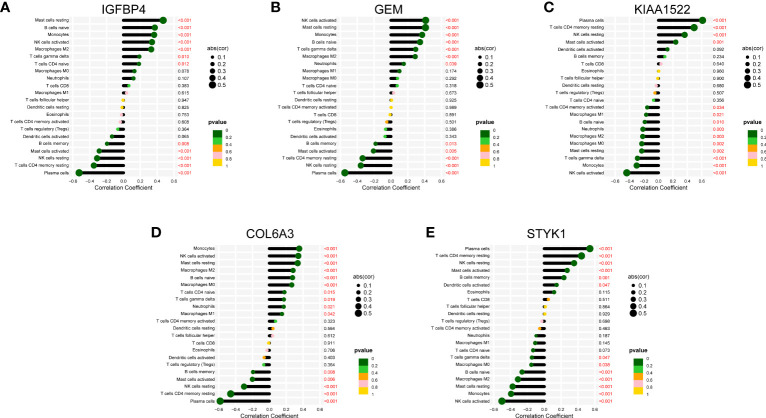
Correlation map of 22 types of immune cells and 5 selected genes. A positive and negative correlation was respectively shown in the right and left direction, whereas the size of the circle represents the strength of correlation, the larger the size, the stronger the correlation. **(A)** IGFBP4. **(B)** GEM. **(C)** KIAA1522. **(D)** COL6A3. **(E)** STYK1.

**Figure 9:**
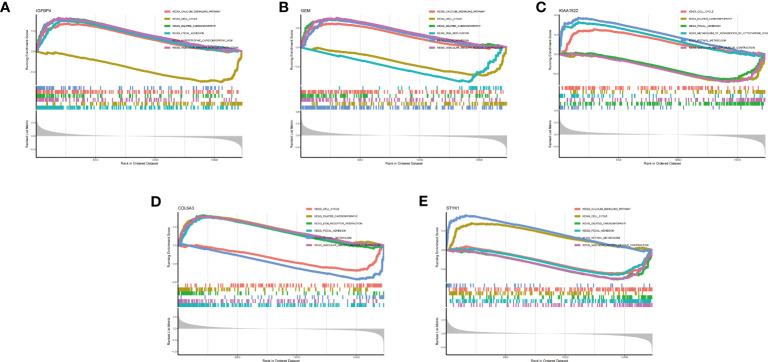
GSEA analysis of 5 selected genes. **(A)** IGFBP4. **(B)** GEM. **(C)** KIAA1522. **(D)** COL6A3. **(E)** STYK1.

We also discussed the associations between 5 characteristic genes and distinct immune cell types, the findings revealed that IGFBP4 had positive associations with resting mast cells, naïve B cells, monocytes, activated NK cells, M2 Macrophages, γδ T cells, naïve CD4 T cells, and negative associations with memory B cells, activated Mast cells, resting NK cells, resting CD4 memory T cells, and plasma cells (**Figure 8A**). Similarly, a significant correlation was also observed between additional biomarkers (GEM, GEM, KIAA1522, COL6A3, and STYK1) and infiltrating immune cells (**Figures 8B–E**). These findings demonstrated some kinds of immune cells are dysregulated in the progression of GC. Additionally, The GSEA analysis results showed that the cell cycle and focal adhesion were crucial biological activities in the development of GC (**Figures 9A–E**), consistent with the abovementioned results.

## Discussion

At present, the diagnosis of GC has relied on gastroscopy and pathological judgment of biopsy tissue, and these methods are limited by their complexity and invasiveness (13, 37) with the development of high-throughput histology technology, systematically identifying the molecular biomarkers of GC is a promising approach from multi-omics levels (38). Therefore, the identification of more valuable molecular patterns is critical to guide the personalized treatment of GC. PANoptosis is a harmonious network where the three programmed cell death-related pathways can be surrogates for each other and interact together in response to tumor microenvironment stimulation (39, 40). However, the specific functions of PANoptosis and its relevant mechanisms in different diseases have not been sufficiently explored. Therefore, we aimed to discuss the detailed role of PRGs in GC phenotyping and the immune microenvironment. Furthermore, gene signatures associated with PANoptosis were applied for predicting the GC patterns.

In this work, we comprehensively discussed the expression characteristics of PANoptosis modulators between normal samples and GC patients. The dysregulated PRGs were observed in GC patients more than those in control samples, indicating an essential part of PRGs in the development of GC. Afterward, we assessed the relationship among PRGs to determine the association between PANoptosis modulators and GC. We found that some PANoptosis regulators demonstrated considerable interactions, as proven by the presence of PRG interactivity in GC patients. Meanwhile, the infiltrating levels of immune cells were different between control samples and GC patients. GC patients presented greater abundant ratios of naïve B cells, naïve CD4 T cells, activated memory CD4 T cells, follicular helper T cells, activated NK cells, M0 Macrophages, M1 Macrophages, and Neutrophils, consistent with the prior reports. Moreover, we employed an unsupervised cluster approach to present the distinct PANoptosis regulation patterns in GC patients according to the expression status of PRGs, and two different PANoptosis -related patterns were determined. Cluster-specific DEGs demonstrated that Cluster1 was largely engaged in immune-related biological processes, such as TGF-b signaling and Notch signaling pathway, while Cluster2 was featured by metabolic activity. Consistently, Cluster1 had a greater level of Chemokine signaling and JAK-STAT signaling. Therefore, it would be valuable to consider that Cluster1 may present more activated B cells and T cells to eliminate the development of GC and further demonstrate a better prognosis.

The available machine learning algorithms enable bioinformatic technology to more accurately and rapidly identify promising biomarkers correlated with disease initiation and development, allowing for disease diagnosis, treatment, and therapeutic agents’ investigation (41). In this work, we examined the predictive ability of four machine learning algorithms (RF, SVM, GLM, and XGB) predicated on the expression level of cluster-specific DEGs and constructed an SVM-based prediction signature, which demonstrated the greatest predictive performance in the testing cohort, indicating the SVM-based machine learning signature has promising properties in depicting the patterns of GC. Afterward, five special variables (IGFBP4, GEM, KIAA1522, COL6A3, and STYK1) were selected to establish an SVM-based model. The 5-gene-based SVM model can reliably predict GC in the external cohorts (AUC = 0.970 and 0.916), which gives new perspectives for the diagnosis of GC. Moreover, we also established a nomogram model for the diagnosis of GC patterns with these 5 predictor genes, and this model presented dramatic predictive value, suggesting the significance of this predictive model for clinical utilities.

As the most prevalent IGFBP in circulation, IGFBP4 has been reported to serve a crucial role in tumor development regulation by inhibiting IGF activities (42). Bioinformatics analysis presented that IGFBP4 could play a critical biomarker and prognostic predictor for GC (43). GEM is a regulative protein that might be involved in the receptor-activated signaling pathway at the plasma membrane, thus serving a pivotal role in fundamental cellular actions (44). Dysregulated KIAA1522 might facilitate the carcinogenesis and metastasis of diverse gastrointestinal tumors through different signaling pathways, such as the Notch signaling pathway (45, 46). And KIAA1522 serves a tumorigenic function in the metastasis of gastrointestinal tumors and might be a promising molecular target for further management. COL6A3 has been reported to be participated in the development of GC by modulating the PI3K/AKT signaling pathway (47), and some research demonstrated that inhibition of COL6A3 would make a great prognosis in GC patients (48, 49). Low STYK1 expression presented an unfavorable prognosis for GC, and STYK1 could be a diagnostic and prognostic predictor in GC patients (50).

Currently, PANoptosis can stimulate powerful anti-tumor immunity, and facilitating PANoptosis could have remarkable therapeutic significance in GC (51). Meanwhile, accumulative studies have observed that immunological disorders are essential pathobiological factors responsible for the proliferation and unfavorable prognosis of GC (52, 53). Therefore, we conducted a correlation analysis between these selected PANoptosis pattern-related genes with infiltrating immune cells. The findings indicated differential levels of Plasma cells, resting CD4 memory T cells, Monocytes, resting and activated NK cells, M2 Macrophages, naive B cells, resting and activated Mast cells, and γδ T cells between control and GC. Plasma cells, naive B cells, and Mast cells participated in antibody production and further mediated the tumor progression (54–56). The positive activity of NK cells is responsible for the therapeutic outcome and prognostic significance of GC, while the volume and spread of the tumor also affect the NK cell functions in patients (57). Polarization of M2 macrophages in the GC microenvironment could stimulate tumor metastasis by promoting the EMT process in GC (58). γδ T cells in GC tissue can regulate gastric carcinogenesis by secreting different cytokines (59). Moreover, resting CD4 memory T cells modulated immunological status, and Monocytes mediated prognostic evaluation are reported from prior studies during the GC (60, 61).

Inevitably, there are certain deficiencies and limitations in this research. The findings of our study should be further confirmed *in vivo* or *in vitro* studies. Also, the prognostic value of this SVM-based model is pending to be evaluated in the external database with complete survival information. Therefore, further experimental and prospective studies are necessary in the future.

## Conclusion

In summary, this work revealed the association between PRGs and immune cell infiltration and demonstrated the considerable immunological heterogeneity between GC patients with different PANoptosis patterns. A 5-gene-based SVM model was identified as the valuable machine learning signature, which can reliably evaluate GC patterns and the pathophysiologic process of GC patients. Our research provides the first identification of the potential value for PANoptosis in GC and reveals the potential molecular characteristics contributing to GC heterogeneity, and improve personalized therapy in GC based on PANoptosis patterns.

## Data availability statement

The datasets presented in this study can be found in online repositories. The names of the repository/repositories and accession number(s) can be found in the article/**Supplementary Material**.

## Author contributions

All authors contributed to the study’s conception and design. XQ performed data collection and analysis. XQ and JJ wrote the manuscript. KW, KX, and CY polished and revised the manuscript. All authors contributed to the article and approved the submitted version.

## Supplementary material

Supplementary Figure 1 Biological characteristics between two PANoptosis clusters. **(A)** Differences in hallmark pathway activities between Cluster1 and Cluster2 samples ranked by t-value of GSVA method. **(B)** Differences in KEGG pathways between Cluster1 and Cluster2 samples ranked by t-value of GSVA method.

Supplementary Figure 2 Immunological characteristics between two PANoptosis clusters. **(A, B)** Differences in HLA and immune-checkpoint molecules between Cluster1 and Cluster2. **(C)** Differences in immune activity between Cluster1 and Cluster2.
