# Supplementary figures and images for: Expression patterns and immunological characterization of PANoptosis -related genes in gastric cancer
Source: Front Endocrinol (Lausanne). 2023 Aug 18;14:1222072. doi: 10.3389/fendo.2023.1222072 (PMC10471966; doi:10.3389/fendo.2023.1222072)

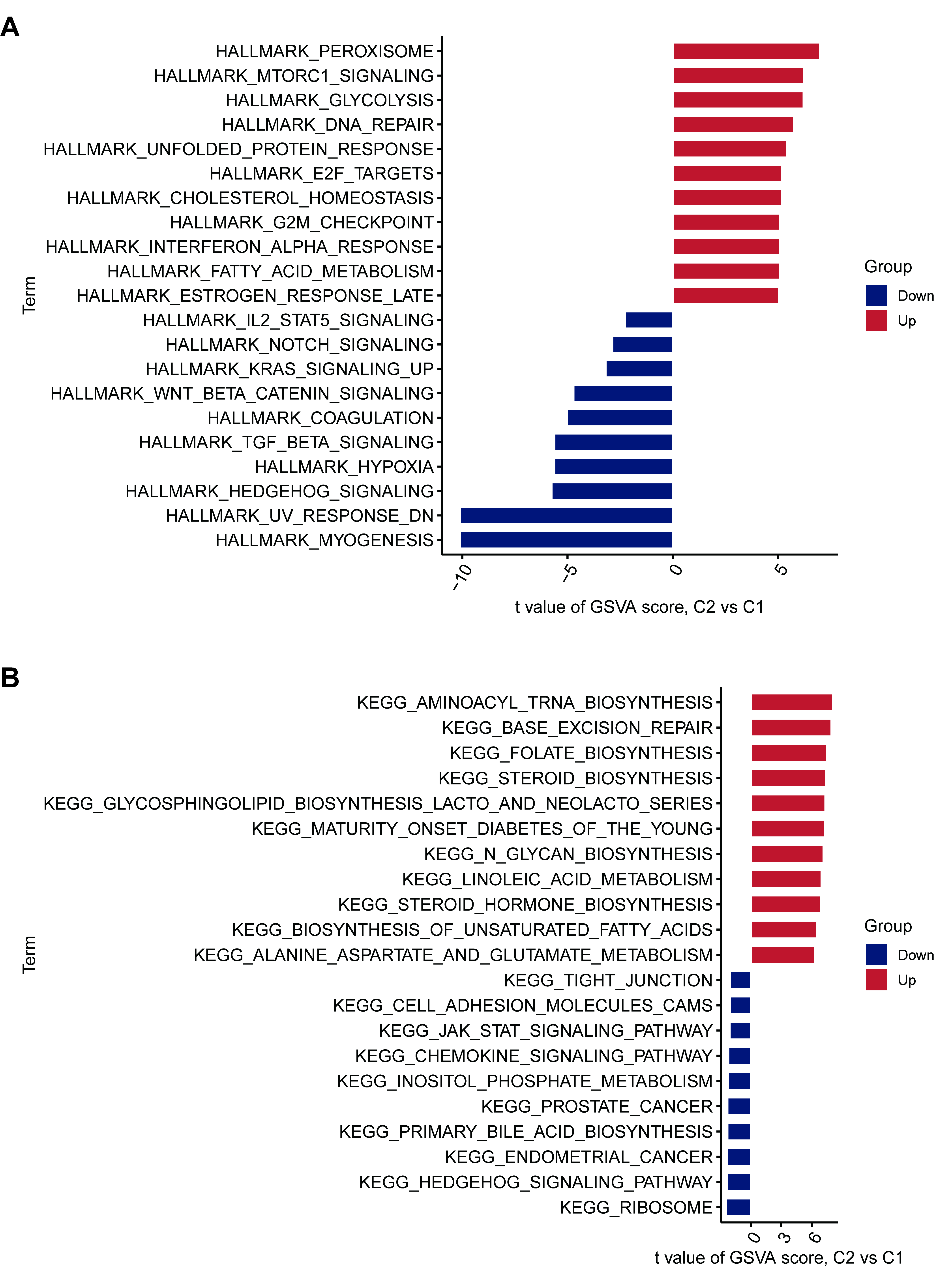

Supplement: Supplementary Figure 1 — Biological characteristics between two PANoptosis clusters. (A) Differences in hallmark pathway activities between Cluster1 and Cluster2 samples ranked by t-value of GSVA method. (B) Differences in KEGG pathways between Cluster1 and Cluster2 samples ranked by t-value of GSVA method. [file Image_1.tif]

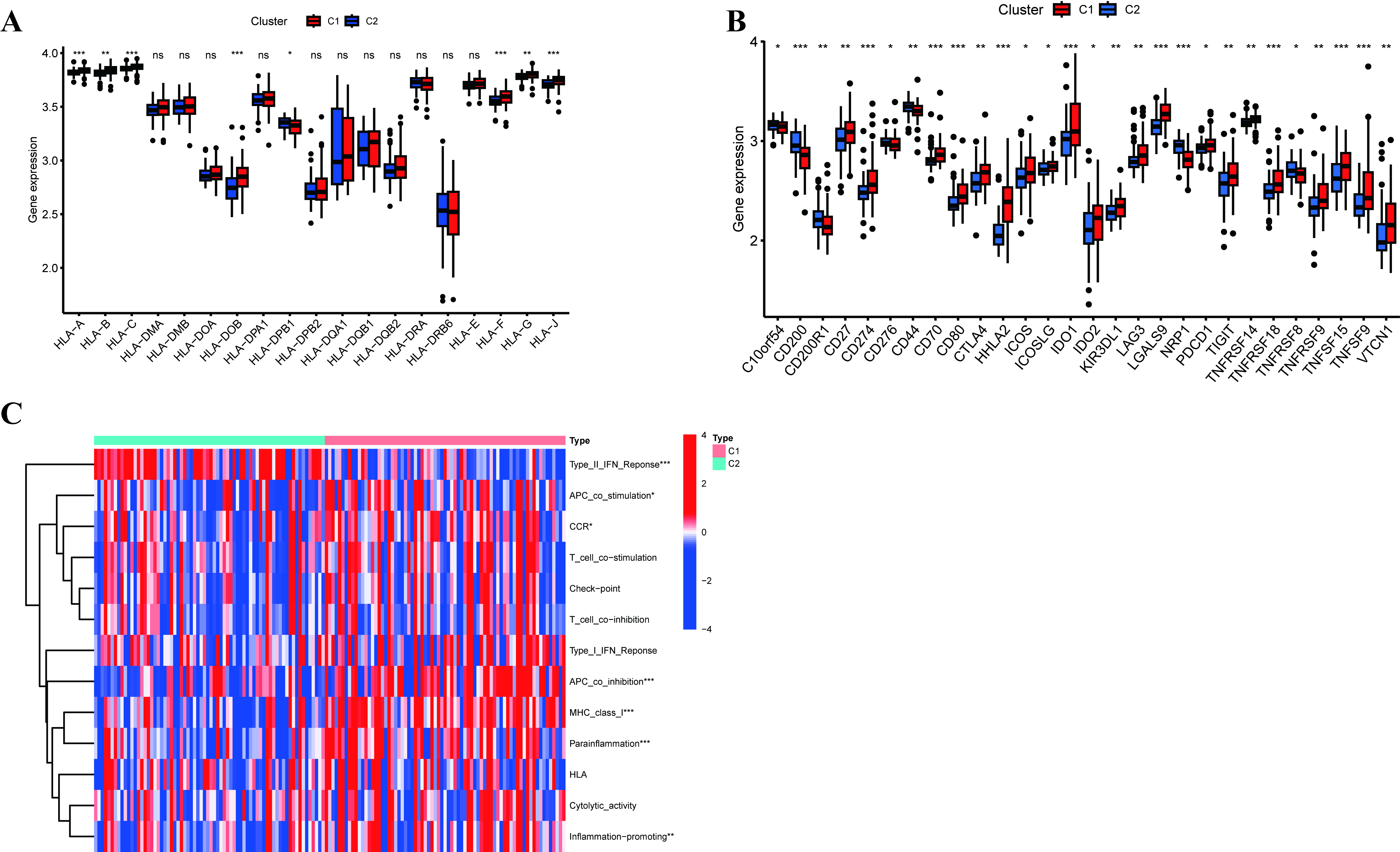

Supplement: Supplementary Figure 2 — Immunological characteristics between two PANoptosis clusters. (A, B) Differences in HLA and immune-checkpoint molecules between Cluster1 and Cluster2. (C) Differences in immune activity between Cluster1 and Cluster2. [file Image_2.tif]
